# Mesenchymal stem cells confer chemoresistance in breast cancer via a CD9 dependent mechanism

**DOI:** 10.18632/oncotarget.26952

**Published:** 2019-05-28

**Authors:** Mujib Ullah, Asma Akbar, Nathan Norton Ng, Waldo Concepcion, Avnesh S. Thakor

**Affiliations:** ^1^ Interventional Regenerative Medicine and Imaging Laboratory, Stanford University School of Medicine, Department of Radiology, Palo Alto, CA 94304, USA; ^2^ Mid-Florida Research and Education Center, Department of Pathology, University of Florida, Apopka, FL 32703, USA

**Keywords:** MSCs, CD9, chemoresistance, cytokine, xenograft tumors

## Abstract

The development of chemotherapy drug resistance remains a significant barrier for effective therapy in several cancers including breast cancer. Bone marrow-derived mesenchymal stem cells (BMMSCs) have previously been shown to influence tumor progression and the development of chemoresistance. In the present study, we showed that when GFP labelled BMMSCs and RFP labelled HCC1806 cells are injected together *in vivo*, they create tumors which contain a new hybrid cell that has characteristics of both BMMSCs and HCC1806 cells. By labelling these cells prior to their injection, we were then able to isolate new hybrid cell from harvested tumors using FACS (DP-HCC1806:BMMSCs). Interestingly, when DP-HCC1806:BMMSCs were then injected into the mammary fat pad of NOD/SCID mice, they produced xenograft tumors which were smaller in size, and exhibited resistance to chemotherapy drugs (i.e. doxorubicin and 5-fluorouracil), when compared tumors from HCC1806 cells alone. This chemoresistance was shown to associated with an increased expression of tetraspanins (CD9, CD81) and drug resistance proteins (BCRP, MDR1). Subsequent siRNA-mediated knockdown of BMMSC-CD9 in DP-HCC1806:BMMSCs resulted in an attenuation of doxorubicin and 5-fluorouracil chemoresistance associated with decreased BCRP and serum cytokine expression (CCL5, CCR5, CXCR12). Our findings suggest that within the tumor microenvironment, CD9 is responsible for the crosstalk between BMMSCs and HCC1806 breast cancer cells (via CCL5, CCR5, and CXCR12) which contributes to chemoresistance. Hence, BMMSC-CD9 may serve as an important therapeutic target for the treatment of breast cancer.

## INTRODUCTION

Breast cancer is the most diagnosed cancer and leading cause of cancer related death amongst women, with a global incidence of nearly 1.7 million new cases each year and over 520,000 deaths [1, 2]. It is categorized according to several subtypes and is highly heterogeneous in its disease progression, rate of metastasis, and prognosis, thereby making it a challenge to treat [3]. While chemotherapy is part of the standard of care for patients with breast cancer, the development of drug resistance remains a significant barrier for effective therapy [4]. Hence, uncovering the mechanisms that promote chemoresistance is important for developing therapies that can treat breast cancer by impeding tumor growth as well as preventing disease relapse.

The progression of breast cancer and its subsequent development of chemoresistance, is highly dependent on the paracrine and cell-cell interactions between the tumor and its surrounding microenvironment, which consists of fibroblasts, immune cells, endothelial cells, and mesenchymal stem cells (MSCs) [5]. MSCs are self-renewing multipotent cells, found in bone marrow, adipose tissue, umbilical cord blood, and placental tissue, that are capable of differentiating into cells of the mesodermal lineage such as adipocytes, chondrocytes, and osteocytes [6–8]. Although MSCs have an important well-defined therapeutic role in tissue repair and regenerative medicine [9–12], their role in cancer biology is less certain. While MSCs have been shown to be recruited to the site of tumors, via endocrine and paracrine signaling [13–15], there are multiple studies reporting their anti, as well as pro-, tumorigenic properties. Indeed, some studies have shown that MSCs can promote tumor progression by stimulating tumor proliferation, angiogenesis, motility, metastasis, and tissue invasion [16–19]. In contrast, other studies have shown MSCs to have an inhibitory effect on cancer progression by inducing apoptosis, suppressing signaling pathways, initiating cell-cycle arrest, and increasing infiltration of monocyotes and granulocytes [20–23].

Hence, further clarification of the molecular interactions between different cancer subtypes and MSCs is warranted. In the present study, we investigated the effect of human bone marrow-derived MSCs (BMMSCs) on an ERα-, PR-, and HER2-negative (triple negative) breast cancer cell line (HCC1806). We found that BMMSCs and HCC1806 cells behave and interact differently depending on whether they are co-cultured *in vitro* or *in vivo*. Indeed, we found that when BMMSCs and HCC1806 cells are co-cultured *in vivo*, they create tumors which contain a new hybrid cell (DP-HCC1806:BMMSCs) that has characteristics of both BMMSCs and HCC1806 cells. Following isolation of DP-HCC1806:BMMSCs, we then showed *in vivo* that this hybrid cell could create tumors like conventional HCC1806 cells, but that these tumors were reduced in size and exhibited increased resistance to chemotherapeutic agents. Next, we determined that this effect was dependent on the expression of CD9 in BMMSCs. Taken together, our findings provide insight into the possible mechanism by which BMMSCs may influence breast cancer development and chemotherapy drug resistance.

## RESULTS

### Determining the interaction between BMMSCs and HCC1806 breast cancer cells

**Figure 1:**
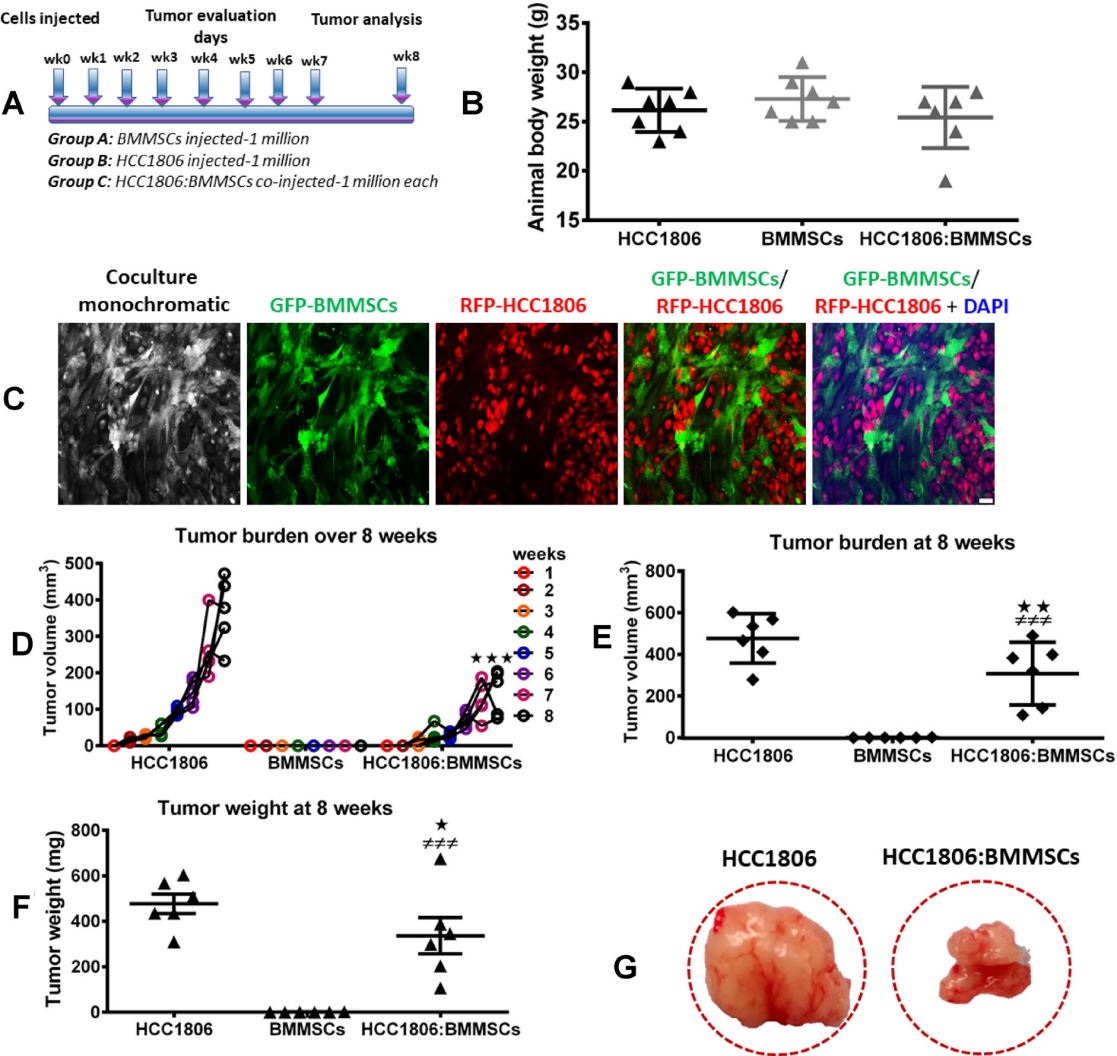
BMMSCs reduce tumor burden of HCC1806 xenografts *in vivo*. (**A**) Schematic diagram of our experimental design. Xenografts consisting of BMMSCs (1 × 10^6^ cells), HCC1806 cells (1 × 10^6^ cells), or a coculture of HCC1806:BMMSCs (1 × 10^6^ cells per line) were injected into immunocompromised NOD/SCID mice. Tumor burden was assessed weekly in injected animals, for a total of 8 weeks. (**B**) Total animal body weight by week 8. (**C**) Representative images of bone marrow-derived BMMSCs, labeled with GFP, and HCC1806 breast cancer cells, labeled with RFP, that were cocultured prior to *in vivo* injection. (**D**) Weekly tumor volume in grafted animals over 8 weeks. (**E**) Week 8 tumor volume in excised tissue samples. (**F**) Week 8 tumor weight in excised tissue samples. (**G**) Representative images of tumor excised from sacrificed animals. Significance indicated by ^*^p < 0.05, ^**^p < 0.01 for comparison between HCC1806 and HCC1806:BMMSCs. ^###^*p* < 0.001 for comparison between BMMSCs and HCC1806:BMMSCs. Scale bar 100 μm.

**Figure 2:**
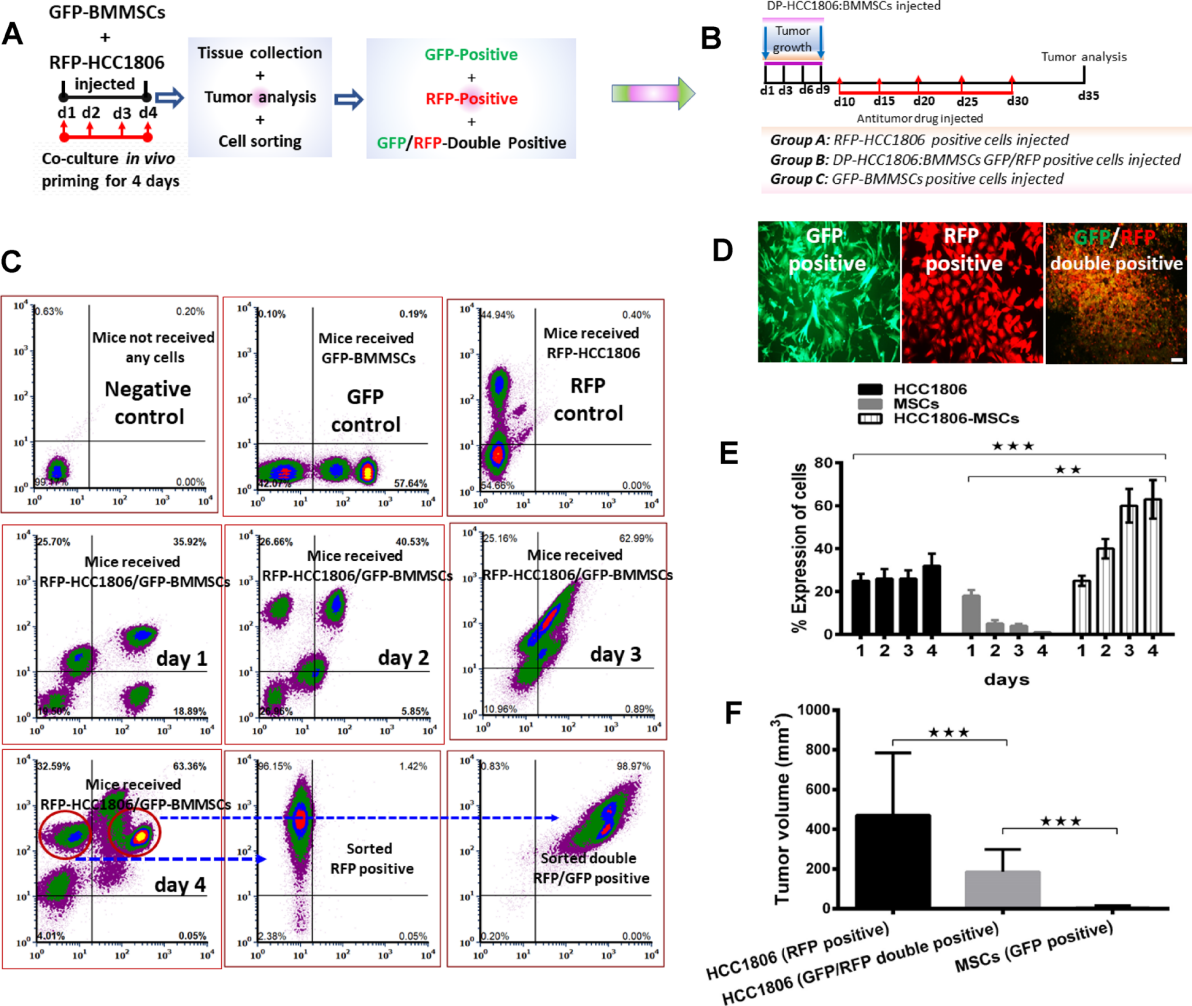
FACS-sorted GFP/RFP-double positive cells from HCC1806:BMMSC xenografts reduced tumor volume *in vivo*. (**A**) Animals injected with MSC-GFP, HCC1806-RFP, and HCC1806-RFP:MSC-GFP xenografts are monitored over a period of four days. On each day, xenografts are harvested and FACS-sorted into GFP-positive (BMMSCs), RFP-positive (HCC1806 cells), or GFP/RFP-double positive cells (DP-HCC1806:BMMSCs). (**B**) Schematic diagram of our experimental design: Sorted cells are reinjected into the animals to assess tumor burden. (**C**) FACS sorting of GFP-positive, RFP-positive, and GFP/RFP-double positive cells from xenografts isolated from vehicle, GFP-BMMSC, RFP-HCC1806, and DP-HCC1806:BMMSC xenografted animals (*n* = 6 animals). (**D**) Representative micrographs of FACS-sorted GFP-BMMSCs, RFP-HCC1806 cells, and GFP/RFP-double positive cells. (**E**) Percent total expression of cells sorted from harvested xenografts during a four day period. (**F**) Animals were reinjected with either FACS-sorted RFP-positive cells (HCC1806 cells), GFP/RFP-double positive cells (DP-HCC1806:BMMSCs), or GFP-positive cells (BMMSCs), and tumor volume was assessed at day 35 post-injection. Data are reported as mean ± s.e.m., with significance indicated by ^***^p < 0.001 and ^**^p < 0.01. Scale bar 100 μm.

To evaluate the effect and interaction between RFP-labeled HCC1806 cells and GFP-labeled BMMSCs, these cells were injected either alone or together into the mammary fat pad of NOD/SCID mice (Figure 1A–1C). While BMMSCs did not grow into tumors in this environment, RFP-labeled HCC1806 cells did produce tumors which progressively increased in size over 8 weeks (Figure 1D). Interestingly, when RFP-labeled HCC1806 cells and GFP-labeled BMMSCs were injected together, the tumor size was markedly reduced in comparison to tumors created by RFP-labeled HCC1806 cells (Figure 1E–1G). To further evaluate the interaction between RFP-labeled HCC1806 cells and GFP-labeled BMMSCs, both cells were injected *in vivo* with tumors isolated daily from animals over the next 4 days. The different cell populations were then sorted through a fluorescence activated cell sorter (FACS) (Figure 2A–2C). While there was no significant change in the percent expression of RFP-HCC1806 cells over 4 days, there was a steady decrease in the percent expression of GFP-BMMSCs and this was accompanied by an increase in the percent expression of a new population of HCC1806:BMMSCs (i.e. hybrid cells which were double positive (DP) for GFP-BMMSCs and RFP-HCC1806 cells) (Figure 2D, 2E). This new population of cell: DP-HCC1806:BMMSCs, was then specifically examined in all of our subsequent experiments.

### Evaluating the *in vivo* effect of BMMSCs in xenograft breast cancer animal models

In NOD/SCID mice, the following cells were injected into the mammary fat pad: RFP-HCC1806 cells, GFP-BMMSCs, or DP-HCC1806:BMMSCs. Animals which received GFP-BMMSCs alone produced no tumors, however, animals which received either RFP-HCC1806 alone or DP-HCC1806:BMMSCs developed tumors. At week 8, although there was no difference in the body weight of animals (Figure 1B), the excised tumors from DP-HCC1806:BMMSCs xenograft animals had a decreased volume when compared to animals which received RFP-HCC1806 cells alone (Figure 2F).

### Evaluating the *in vivo* effects of RFP-HCC1806 cells and DP-HCC1806:BMMSCs to chemotherapeutic drugs

**Figure 3:**
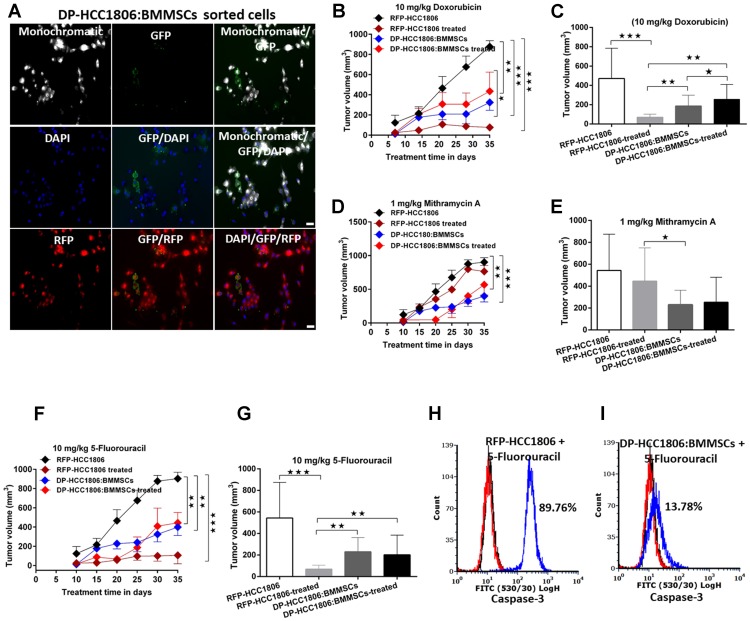
DP-HCC1806:BMMSC xenografts mediate chemotherapeutic resistance. (**A**) Representative images showing the growth of sorted cells. (**B**) Tumor volume upon *in vivo* administration of doxorubicin (10 mg/kg) for 25 days. (**C**) Tumor volume at day 35 in doxorubicin-treated animals. (**D**) Tumor volume upon *in vivo* administration of mithramycin A (1 mg/kg) for 35 days. (**E**) Tumor volume at day 35 in mithramycin A-treated animals. (**F**) Tumor volume upon *in vivo* administration of 5-fluorouracil (5FU) (10 mg/kg) for 35 days. (**G**) Tumor volume at day 35 in 5FU-treated animals. (**H**, **I**) *In vivo* tumor reduction validated by flow cytometric confirmation of caspase-3 cell death assays in HCC1806 and DP-HC1806:BMMSCs xenografted animals, *n* = 6 animals). Data are reported as mean ± s.e.m., with significance indicated by ^***^p < 0.001, ^**^p < 0.01 and ^*^p < 0.05. Scale bar 100 μm.

In NOD/SCID mice, the following cells were injected into the mammary fat pad: RFP-HCC1806 cells or DP-HCC1806:BMMSCs. After 10 days, animals were subjected to 25 days of chemotherapy (i.e. either doxorubicin (Dox; 10 mg/kg), mithramycin A (MTR; 1 mg/kg), or 5-fluorouracil (5FU; 10 mg/kg). In xenograft animals created with RFP-HCC1806 cells, there was a reduction in both the rate and magnitude of tumor growth when animals were treated with Dox or 5FU compared to control animals which received no chemotherapy treatment. In contrast, in xenograft animals created with DP-HCC1806:BMMSCs, treatment with Dox and 5FU resulted in no change in the rate and magnitude of tumor growth compared to control animals, thereby demonstrating chemoresistance within these animals (Figure 3A–3C, 3F, 3G). The limited reduction in tumor volume in xenograft animals created with DP-HCC1806:BMMSCs was also accompanied by a reduction in caspase-3 activity following 5FU treatment (Figure 3H–3I). Regardless of the cells used to create xenograft animals, there was no difference in either the rate or magnitude of tumor growth when animals were given MTR compared to animals which received no chemotherapy treatment (Figure 3D, 3E).

### Determining the mechanism of chemoresistance of DP-HCC1806:BMMSCs to Dox and 5FU

**Figure 4:**
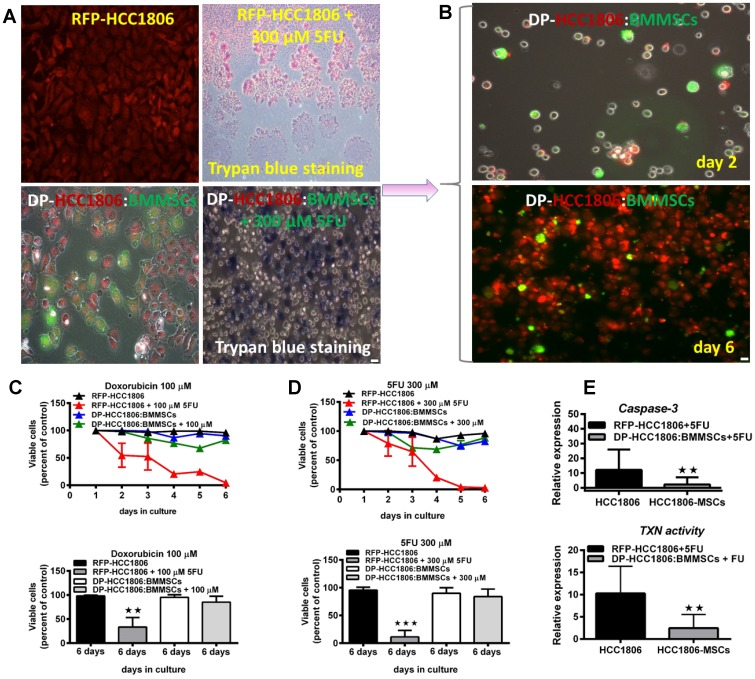
Increased cell viability and reduced cytotoxicity underlie chemoresistance to doxorubicin and 5FU. (**A**, **B**) *In vitro* cell death assessed qualitatively using trypan blue exclusion in HCC1806 and HCC1806:BMMSCs cocultures treated with either 300 μM 5FU or 100 μM doxorubicin for 6 days. (**C**) MTT assay for doxorubicin-treated cultures and percent viable cells by day 6 of treatment. (**D**) MTT assay for 5FU-treated cultures and percent viable cells by day 6 of treatment. (**E**) Degree of *in vitro* cytotoxicity to chemotherapeutic agents, assessed through caspase-3 and thioredoxin (TXN) reductase activity. Data are reported as mean ± s.e.m., with significance indicated by ^**^p < 0.01, ^***^p < 0.001. Scale bar 100 μm.

**Figure 5:**
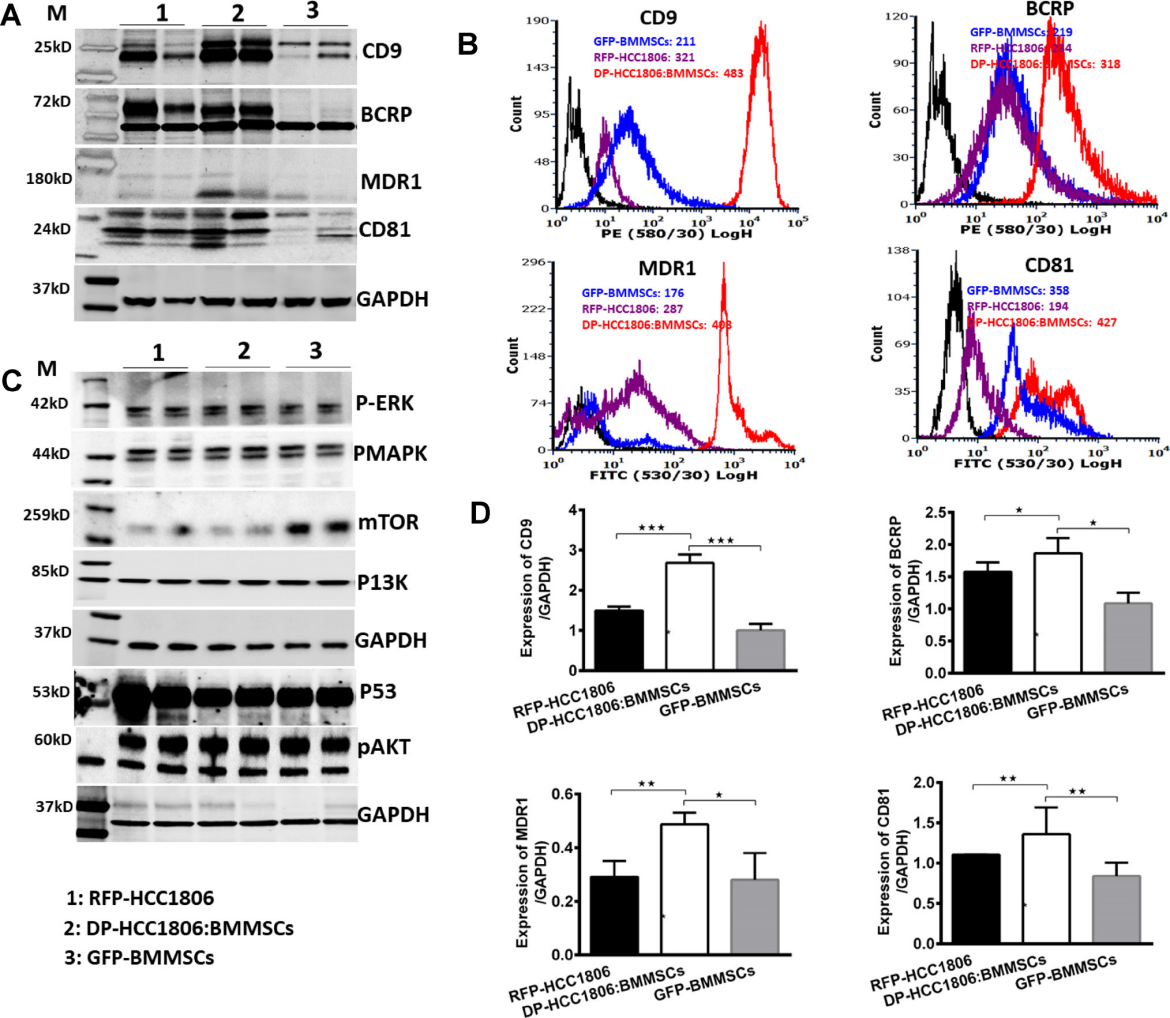
Differential expression of tetraspanins and drug resistance proteins in DP-HCC1806:BMMSCs. (**A**) Western blot analyses of sorted cells for CD9, BCRP, MDR1 and CD81 expression. (**B**) Flow cytometry analysis of CD9, BCRP, MDR1, and CD81 protein expression distribution. (**C**) Western blot analyses of sorted cells for p-ERK, pMAPK, mTOR, PI3K, p53, and pAKT expression. (**D**) Quantification of western blot data proteins confirming increased expression of proteins CD9, BCRP, MDR1 and CD81 protein. Data are reported as mean ± s.e.m., with significance indicated by ^*^p < 0.05, ^**^p < 0.01, ^***^p < 0.001.

Following 6 days of *in vitro* culture, RFP-HCC1806 cells or DP-HCC1806:BMMSCs were treated with either Dox (100 μM) or 5FU (300 μM). From day 2 to day 6, chemotherapy treatment resulted in RFP-HCC1806 cells demonstrating increased cell apoptosis (assessed qualitatively using Trypan blue exclusion (Figure 4A, 4B)); reduced cell viability (assessed quantitatively using an MTT assay (Figure 4C, 4D)); and increased cell proliferation (assessed quantitatively using RFP/GFP fluorescence (Figure 4B)). In contrast, by day 6, DP-HCC1806:BMMSCs demonstrated no significant change in cell viability or proliferation following chemotherapy treatment. This was accompanied by DP-HCC1806:BMMSCs showing reduced expression of cytotoxic caspase-3 and thioredoxin reductase when compared to RFP-HCC1806 cells (Figure 4E). To further investigate the mechanisms underlying the chemoresistance of DP-HCC1806:BMMSCs, we evaluated the changes in the expression pattern of tetraspanin proteins (CD9 and CD81), drug resistance proteins (BCRP and MDR1), and common targets of cancer pathways (p-ERK, pMAPK, mTOR, PI3K, pAKT, and p53) (Figure 5A–5D). Western blot analysis demonstrated an increase in the protein expression of CD9, CD81, BCRP and MDR1 accompanied by a decrease in the protein expression of mTOR in DP-HCC1806:BMMSCs compared to both GFP-BMMSCs or RFP-HCC1806 cells. Flow cytometric distribution analyses indicated that the greatest change was in the expression of CD9 and MDR1 (Figure 5B).

### The role of CD9 in mediating the chemoresistance of DP-HCC1806:BMMSC

**Figure 6:**
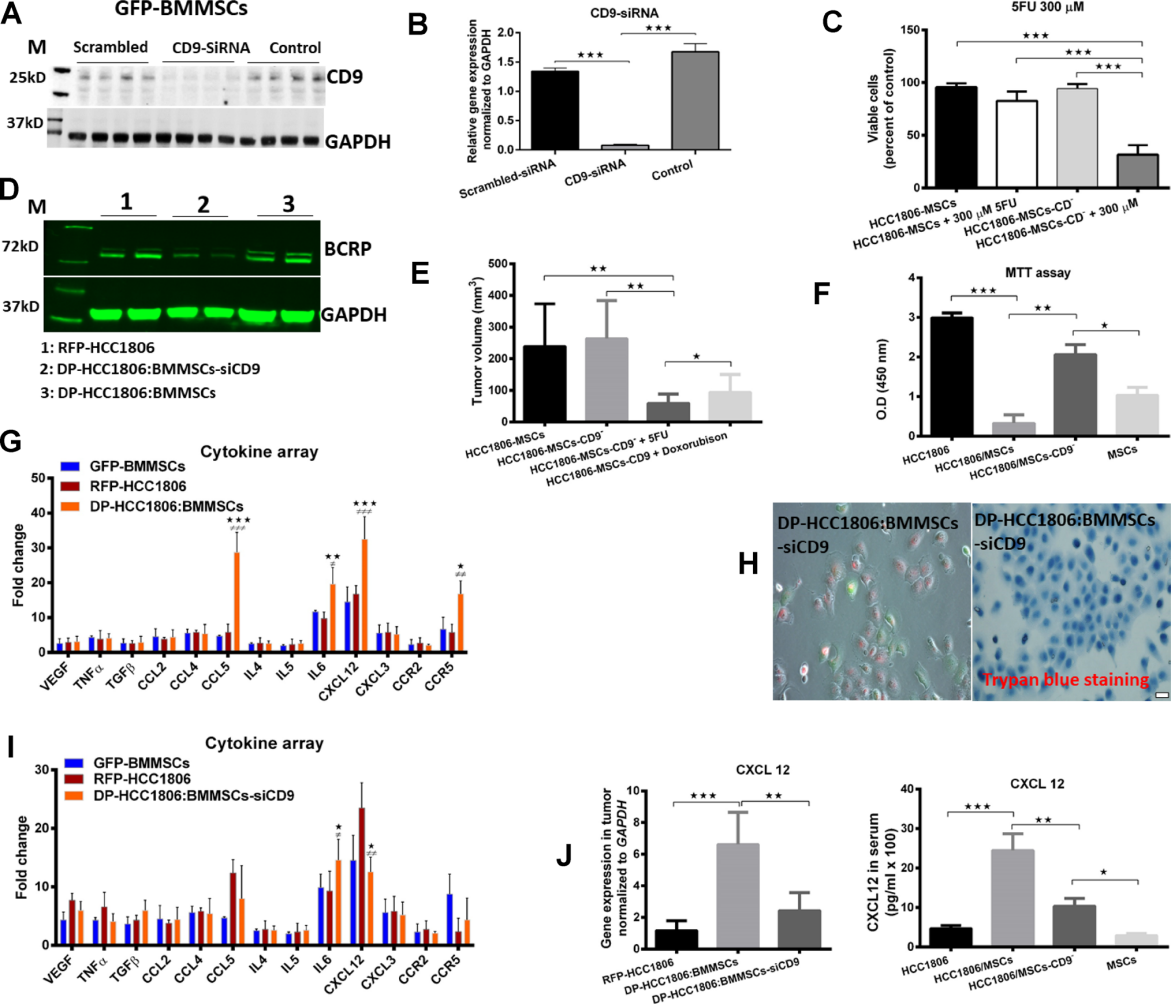
BMMSCs-CD9 siRNA knockdown in DP-HCC1806:BMMSC xenografts reduces chemotherapeutic resistance to 5FU and doxorubicin *in vivo*. (**A**, **B**) Western blot and real time PCR analyses confirming siRNA-mediated knockdown of CD9 in BMMSCs. (**C**) Percent viability of 5FU-treated DP-HCC1806:BMMSCs and DP-HCC1806:BMMSCs-siCD9 cultures. (**D**) Western blot analyses of BCRP expression. (**E**) *In vivo* tumor volume in animals co-injected with DP-HCC1806:BMMSCs or DP-HCC1806:BMMSCs-siCD9 cells, evaluated at week 4 post-injection. (**F**) MTT assay for BMMSC, HCC1806, DP-HCC1806:BMMSCs, and DP-HCC1806:BMMSCs-siCD9 cultures. (**G**) Serum levels of various cytokines, and inflammatory factors from mice injected with HCC1806, BMMSCs, and DP-HCC1806:BMMSC xenografts. (**H**) *In vitro* cell death assessed qualitatively using trypan blue exclusion. (**I**) Cytokine profile for BMMSCs, HCC1806 cells, and DP-HCC1806:BMMSCs-siCD9 cells. (**J**, right) Cytokine ELISA assay and real-time RT-PCR analyses of CXCL12 protein and mRNA levels in excised tumor tissue. Mean ± s.e.m, *n* = 6. Significant difference indicated by ^*^p < 0.05, ^**^p < 0.01, ^***^p < 0.001 for comparison between HCC1806 and DP-HCC1806:BMMSCs. ^#^*p* < 0.05, ^##^*p* < 0.01, ^###^*p* < 0.001 for comparison between BMMSCs and DP-HCC1806:BMMSCs. Scale bar 100 μm.

Next, we performed siRNA-mediated knockdown of CD9 in BMMSCs and then co-cultured these cells *in vivo* with HCC1806 (Figure 6A, 6B). DP-HCC1806:BMMSCs-siCD9 were then isolated as previously described using FACS. When DP-HCC1806:BMMSCs-siCD9 were used to create a xenograft model, tumors were now responsive to chemotherapy treatment with both Dox and 5FU showing a reduction in tumor volume at 4 weeks when compared to the same treatment given to xenograft animals created with DP-HCC1806:BMMSCs (Figure 6E). *In vitro* analysis of DP-HCC1806:BMMSCs-siCD9 also now demonstrated these cells to now be sensitive to chemotherapeutics with them exhibiting a decrease in cell viability (assessed quantitatively using an MTT assay) and increased apoptosis (assessed quantitatively using Trypan blue exclusion), compared to DP-HCC1806:BMMSCs after 6 days of treatment (Figure 6C, 6F, 6H). This reduced viability was also accompanied by a decrease in BCRP protein expression in DP-HCC1806:BMMSCs-siCD9 cells at day 6 (Figure 6D).

### Analysis of serum and tissue cytokines in xenograft breast cancer animal models

In order to understand the molecular interactions and signaling pathways involved between BMMSCs and HCC1806 cells, we performed a screen for different cell surface proteins, growth factors, chemotactic factors, and inflammatory factors in the serum of mice which had been injected with RFP-HCC1806 cells, GFP-BMMSCs, DP-HCC1806:BMMSCs and DP-HCC1806:BMMSC-siCD9 cells. At 4 weeks, animals which had been injected with DP-HCC1806:BMMSCs showed a higher expression of CCL5, CCR5 and CXCL12 compared to RFP-HCC1806 or GFP-BMMSC xenografts (Figure 6G). However, the level of all 3 of these proteins was reduced in the serum of animals which had been injected with DP-HCC1806:BMMSC-siCD9 cells (Figure 6I). We then examined the tumor specimens at the time of sacrifice and again showed that there was an increased expression of CXCL12 in DP-HCC1806:BMMSCs relative to DP-HCC1806:BMMSC-siCD9 was confirmed using real-time RT-PCR and an ELISA (Figure 6J) Of note, regardless of the cells which were injected, the serum expression of IL-6 was increased.

## DISCUSSION

**Figure 7:**
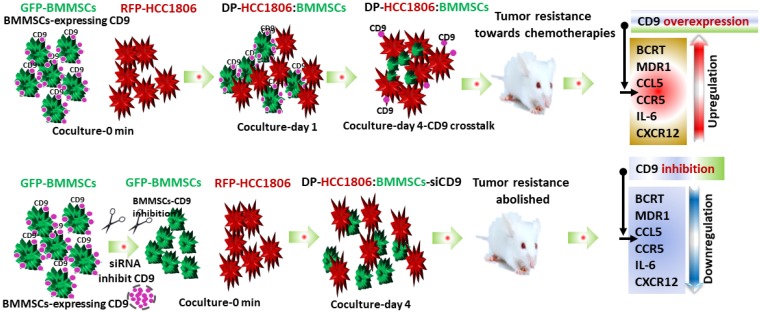
Flow diagram illustrating doxorubicin and 5FU chemoresistance in breast cancer tumors. Schematic diagram showing BMMSCs and HCC1806 coculture. BMMSCs are green due to GFP expression and HCC1806 are red due to RFP expression. *In vivo* co-culture of HCC1806 and BMMSCs cells produced a hybrid cell (DP-HCC1806:BMMSC) whose interaction is dependent on the CD9 expression in BMMSCs. DP-HCC1806:BMMSCs were then used to create xenograft tumors which were smaller but more chemoresistant to agents such as doxorubicin and 5FU. Next, CD9 was inhibited by siRNA in BMMSCs and then co-cultured with HCC1806 cells. These DP-HCC1806:BMMSCs-siCD9 cells were then injected into mice to create xenograft tumors which were now no longer resistant to chemotherapeutic agents. CD9 inhibition also reversed the expression of tumor resistant proteins such as BCRP, CCL5, CCR5, and CXCL12.

In the present study, both RPF-HCC1806 cells and DP-HCC1806:BMMSCs were able to create xenograft tumor models when injected into the mammary fat pad of NOD/SCID mice. However, the tumors created by DP-HCC1806:BMMSCs were smaller as well as more resistant to chemotherapy. Further studies determined that this chemoresistance was due to an increase in expression of CD9, CD81, BCRP and MDR1 proteins. The effects of CD9, which demonstrated the strongest expression in DP-HCC1806:BMMSCs, appeared to be mediated by the CXCL12 protein. When CD9 was silenced in BMMSCs, xenograft animals created with DP-HCC1806:BMMSC-siCD9 now exhibited reduced CXCL12 protein expression with a resulting increased sensitivity to chemotherapy (Figure 7).

For BMMSCs to be considered as a “safe” cell to be used in regenerative medicine, tissue engineering and stem cell therapy applications, it is important to carefully assess whether the cells have any tumorigenic potential. Consistent with previous studies [24–27], our data has shown that BMMSCs are not inherently tumorigenic, especially given that they were not able to induce neoplasms when injected into the mammary fat pad. However, BMMSCs have been shown to facilitate tumorigenic behavior when cultured with breast cancer cells via direct (cell-cell) and indirect (endocrine and paracrine signaling) interactions [16–19, 28, 29]. In support of this, Park *et al.* have reported that while MSCs were not capable of inducing neoplastic transformation, they did however create an inflammatory microenvironment conducive towards tumor growth and angiogenesis [27]. In our studies, we have shown that BMMSCs interact closely with breast cancer cells (i.e. HCC1806), and when co-cultured together *in vivo*, they actually produce a new hybrid cell that has molecular markers of both BMMSCs and HCC1806 cells (DP-HCC1806:BMMSCs); this is likely due to either the BMMSCs being internalized by HCC1806 cells or membrane fragments of BMMSCs being attached to HC1806 cells. Interestingly, this changed the phenotype of HCC1806 cells with the new hybrid DP-HCC1806:BMMSCs producing smaller tumors that were more chemoresistant when injected into the mammary fat pad of animals. Although the exact mechanism by which BMMSCs might be inducing these changes in HC1806 cells is unclear, our work has shown that this may be mediated, in part, by CD9.

CD9 is an integral membrane protein which contains four-membrane spanning domains and is found on the cell surface [30, 31], in exosomes [32, 33], and in nuclei [34]. Studies have shown that CD9 plays a diverse role in both cancer and stem cell biology by regulating numerous cellular processes, such as cell adhesion, proliferation, apoptosis, motility, mitosis, and even extracellular vesicle (EV) secretion [30–38]. In regenerative medicine, activation of BMMSCs increases their CD9 expression which, in turn, has been shown to stimulate their proliferation and regenerative potential [35]. In oncology, CD9 overexpression in tumors has been associated increased risk of invasion and development of metastasis, especially when it forms a complex with its molecular partner CD81, where it then facilitates long-term tumor growth [30, 34]. Interestingly, our results showed that DP-HCC1806:BMMSCs produced smaller tumors; however, these tumors were more chemoresistant to Dox and 5FU which we found was dependent on their increased CD9 expression. In keeping with this, studies in small cell lung cancer (SCLC) cells have shown that ectopic overexpression of CD9 enhances β1 integrin-mediated cell adhesion to extracellular matrix (ECM) fibronectin which has been implicated in cell adhesion mediated drug resistance (CAM-DR) [36, 39]. In addition, there was also a CD9 dependent increase in MDR1 and BCRP; both of which are ABC transporters that have been shown in other cancer models to confer multidrug resistance to chemotherapies such as Dox and 5FU [40, 41]. Although MTR (1 mg/kg) has previously been shown to have anti-tumor effects [42, 43], it had no effect in the present study on xenograft tumors from either RFP-HCC1806 cells or DP-HCC1806:BMMSCs. One explanation for this is that MTR is not able to be transported through MDR1 or BCRP, and has even been shown to downregulate MDR1/BCRP expression [40, 44, 45]. This data therefore suggests that the resistance of RFP-HCC1806 to MTR is independent of CD9-associated BCRP and MDR1 overexpression.

Alternatively, there is also growing evidence to suggest that the chemoresistance of DP-HCC1806:BMMSCs may be due to exosomes given the increased expression of both CD9 and CD81 (Figure 5D), which are two tetraspanins that also happen to be enriched in exosome membranes and serve as exosomal biomarkers [46]. Furthermore, CD9 overexpression been implicated in the increased secretion of extracellular vesicles (EVs), including exosomes [32, 33], and there are studies which have documented acquired chemoresistance of cells through exosome-mediated mechanisms [38, 47]. Koch *et al*. discovered that (i) chemoresistance occurred when B-cell lymphomas sequestered Dox within CD9-positive exosomes which were then exported out of the cell, and (ii) that inhibition of ABC/A3-supported exosome biosynthesis resulted in greater Dox retention within tumor cells [38]. Similarly, Ji *et al*. reported that MSC-derived exosomes conferred drug resistance to 5FU in gastric cancer cells by activating a calcium/calmodulin-dependent protein kinase Raf/MEK/ERK pathway [47]. Based on this literature, it is plausible that the CD9-mediated chemoresistance of DP-HCC1806:BMMSCs to both Dox and 5FU may be due to an increase in BMMSCs-exosome-associated signaling. Hence, future studies will aim to define the exact cellular localization of CD9 within DP-HCC1806:BMMSCs cells.

Given that the inhibition of BMMSC-CD9 expression failed to fully restore chemotherapeutic sensitivity in tumors created from DP-HCC1806:BMMSCs-siCD9 cells, this suggests that other molecular mechanisms may be also contributing towards drug resistance. Although Western blot analyses revealed reduced mTOR protein levels in DP-HCC1806:BMMSCs, there was no significant differences in protein expression of pERK, pMAPK, PI3K, p53, and pAKT, suggesting that these oncogenic pathways (i.e. PI3K/AKT/mTOR and pMAPK/pERK [48, 49]) are not directly involved in mediating the resistance of DP-HCC1806:BMMSCs to Dox and 5FU. However, serum cytokine analyses identified a several fold increase in CXCL12 levels in DP-HCC1806:BMMSCs, which was reduced upon CD9 knockdown, suggesting that CXCL12 expression is correlated with CD9 levels. Interestingly, studies have reported that CD9 and CXCL12 are associated in a CD9/CXCL12/CXCR4 signaling pathway, and activation of this pathway is linked to increased tumor invasion, metastasis, and chemoresistance [50, 51]. Furthermore, in both *in vivo* and *in vitro* models of colorectal cancer, Yu *et al*. reported that activation of CXCL12/CXCR4 conferred miR-125b-mediated resistance to 5FU, causing reduced chemotherapy-induced apoptosis and enhanced autophagy [51].

In animals injected with DP-HCC1806:BMMSCs, we also detected a several fold increase in the serum levels of IL6, CCL5, and CCR5. Interestingly, IL6 is a cytokine that has been shown to be secreted from both tumors and BMMSCs [52, 53] in response to β1 integrin adhesion and has been implicated in maintaining CAM-DR via JAK/STAT3 signaling [54–58]. Likewise, the inflammatory chemokine CCL5, interacting primarily with G protein-coupled receptor CCR5, is highly expressed in several cancers, including breast cancer, and has been shown to play an important pro-oncogenic role via immune cell recruitment, tumor growth, chemotaxis, and apoptosis [59–61]. In addition, CCL5 has been shown to increase αvβ3 integrin expression in cancer cells and increase NF-κB-mediated resistance to drug-induced apoptosis, thus facilitating enhanced integrin-mediated tumor invasion and an immunosuppressive, anti-apoptotic tumor microenvironment [60–63]. Taken together, in this study, the acquisition of 5FU and Dox chemoresistance in DP-HCC1806:BMMSCs is likely due to a CAM-DR mechanism, in which CD9, IL6, and CCL5/CCR5 collectively mediate tumor-growth, adhesion and anti-apoptotic signals, via integrin-dependent mechanisms.

Hence, to fully adopt BMMSCs for clinical applications it will be important for future studies to further define the cellular roles through which BMMSCs interact within a tumor microenvironment.

## MATERIALS AND METHODS

### Human BMMSCs

A frozen vial of 1 × 10^6^ passage-2 human BMMSCs was thawed at 37° C and cultured as previously described [64]. Briefly, BMMSCs were plated in complete culture medium consisting of α-minimum essential medium (α-MEM; Gibco), 20% FBS (Atlanta Biologicals), 100 U/mL penicillin (Gibco), 100 μg/mL streptomycin (Gibco), and 2 mM l-glutamine (Gibco) on a 152-cm^2^ culture dish (Corning). After 24 h, cells were washed with PBS, and the adherent viable cells were harvested by using 0.25% trypsin and 1 mM EDTA (Gibco) for 5 min at 37° C. The harvested cells were plated at 1000 cells/cm^2^ in culture dishes and expanded for 7 days until 70–80% confluence. The culture medium was changed every 2–3 days and were cells were kept in incubators at 37° C with 5% CO_2_.

### HCC1806 cells

A triple negative human breast cancer cell line (HCC1806) was purchased from ATCC (Manassas, VA, USA; CRL2335) and cultured in an α-minimum essential medium (α-MEM; Gibco), containing 10% FBS, 2 mM glutamine, 1 mM sodium pyruvate, and 1 mM nonessential amino acids at 37° C in 5% CO_2_. Cells were passaged every 3 days by incubating for 5 minutes at 37° C first in 0.5 mM EDTA dissolved in PBS followed by 0.5% of trypsin. All other cancer cells were expanded in T-175 culture flasks with filter tops (Corning) using cancer growth medium consisting of αMEM, 10% FBS, 100 units/ml penicillin, and 100 μg/ml streptomycin. The medium was changed every 2–3 days. For all experiments, cancer cells were used when they reached 70–80% confluence.

### Interaction between BMMSCs and HCC1806 cells

BMMSCs and HCC1806 cells were labeled with cell fluorescent green and red florescent red proteins, respectively, as previously described [65]. Phase contrast GFP and RFP images were acquired using a Nikon inverted microscope with an epifluorescence attachment. GFP-BMMSCs and RPF-HCC1806 cells were harvested using trypsin/EDTA and collected by centrifugation at 400 × g for 7 min. The cells were then co-cultured together at 37° C for 3 days in a humidified atmosphere with 5% CO_2_. Unless otherwise indicated, co-cultures of GFP-BMMSCs and RFP-HCC1806 cells were prepared by mixing cell suspensions at a 1:1 ratio. The co-cultures were then expanded by plating at 1000 cells/cm^2^ in complete culture medium, except that FBS was reduced to 10%. In some experiments, co-cultures were initiated in the presence of the CD9 siRNA.

### Preparation of single cells from *in vivo* tumors

To collect single cells, tumors were harvested from the mammary fat pad, mechanically processed into small pieces, washed with PBS buffer and filtered using a cellular strainer to remove undigested cellular derbies. The filtered cells were then transferred to 15 ml conical tubes (Falcon), washed with PBS, and centrifuged at 400 × g for 7 min. To obtain a single-cell suspension, tumors were incubated with trypsin/EDTA at 37° C for 10 min followed by 5 min with collagenase. Every 2 minutes, cells were mechanically disrupted by pipetting 5–10 times. When most aggregates were no longer visible, cells were collected by centrifugation at 400 × g for 7 min. Subsequently, cells were passed through a 40–70 μm cell strainer (Falcon) to remove any remaining cell clusters before staining for flow cytometry.

In some experiments, cells obtained from mice after 4 days and small sections of the tissue were suspended in PBS containing 2%FBS and 1mM EDTA at ~5,000 c/μL and incubated with antibodies (BD Biosciences) for 25 min on ice. Samples were then washed twice in PBS/2%FBS/1 mM EDTA and suspended at a concentration of 2 million cells per mL for FACS. The viable RFP-HCC1806 cell population, GFP-BMMSC cell population, and double positive HCC1806/BMMSCs cell population (i.e. HCC1806 cells with BMMSCs fragments, DP-HCC1806:BMMSCs) were then gated and sorted using a cell sorter (Beckman Coulter). The cells collected were centrifuged at 400 × g for 7 min and washed in PBS. In some experiments, combinations of fluorochrome-conjugated monoclonal antibodies or their respective isotype controls were added to the cell suspension at concentrations recommended by the manufacturer (BD Biosciences) and incubated at 4° C in the dark for 30 min. The labeled cells were washed in PBS and then analyzed using a FACS (BD Biosciences). Gating was set to relevant isotype controls (GFP-FL1 and RFP-FL4) and labeled cells for each cell line. A fraction of the sorted cells was also analyzed on the flow cytometer to ensure viability, complete elimination of the unlabeled cells, and for verification of bright BMMSCs or HCC1806 cells populations. The purity of sorted cells was more than 95% by additional flow cytometric analysis. Sorted populations were injected into mice for tumor evaluation or plated for proteomic assay or processed for genomic assays.

### Real-Time RT-PCR

Isolation of total RNA was performed using the RNeasy mini kit (Qiagen). Total RNA concentration was measured using a NanoDrop ND1000 spectrophotometer (BioRad). RNA (1 μg) was reverse transcribed using oligo dT and transcript or first strand cDNA synthesis kit (Roche). RT-qPCR reactions were carried out in a 20-μL reaction volume containing 3 nM of each primer and SYBR Green PCR Mastermix (Applied Biosystems). Real-time RT-PCR was performed for CXCL12, TWIST1, CD9 and GAPDH using Taqman^®^ Gene Expression Assays (Applied Biosystems). A total of 20–40 ng of cDNA was used for each 20 μl reaction. Thermal cycling was performed with a 7900HT System (Applied Biosystems) by incubating the reactions at 95° C for 20 s followed by 40 cycles of 95° C for 1 s and 60° C for 20 s. Data was analyzed with Sequence Detection Software V2.3 (Applied Biosystems) and relative quantities (RQs) were calculated with comparative C_T_ method using RQ Manager V1.2 (Applied Biosystems). If no amplification occurred, a C_T_ value of ~ 40 was used in calculating the RQs.

### RNA interference (siRNA)

CD9 double-stranded synthetic 21-mer RNA oligonucleotides were used at a final concentration of 200 nM with the Lipofectamine transfection reagent. To knock down CD9 expression in BMMSCs, 200 nM CD9-specific small interfering RNA (siRNA) (5′-GACGUACUCGAAACCUUCA-3′) was transfected. Scrambled siRNA was used as a negative control. 3 days after transfection, cells were analyzed for knockdown efficiency by western blot under non-reducing condition. CD9 siRNA oligomer duplexes targeting CD9 were prepared according to the manufacturer’s instructions. For BMMSCs-siRNA transfection, the cells were transfected with an siRNA mixture against human CD9 or control random siRNAs (life technologies) using Lipofectamine RNAiMAX (Invitrogen). The cells were cultured for 3 days, and the gene-silencing effect of the siRNAs was assessed by immunoblotting with anti-CD9 monoclonal antibody (Santa Cruz).

### Western immunoblotting

Tissues or cells were lysed in a lysis buffer containing 25 mM Tris-HCl (pH 7.6), 150 mM NaCl, 1% sodium deoxycholate, and 0.1% SDS supplemented with protease and phosphatase inhibitor tablets (Roche). Lysates were clarified by centrifugation at 10,000 × g for 10 min and the supernatant containing equal amounts of protein were separated by SDS-PAGE, transferred to PVDF membranes, and probed with primary antibodies followed by peroxidase-conjugated secondary antibodies. Protein levels in the samples were determined by BCA assay kit (Thermo Fisher Scientific, Inc). Approximately 20 μg protein was mixed with SDS buffer (Life Technologies) containing mercaptoethanol (Sigma) and heated at 95° C for 5 minutes. Denatured proteins were separated by electrophoresis on polyacrylamide gels (Biorad Gels; Biorad) and transferred to nitrocellulose membranes using the Biorad turbo transfer System (Biorad). Membranes were blocked for 1 h at room temperature with 5% BSA (Theromofisher) in PBS containing 0.1% Tween-20 (PBST, Biorad). After blocking, membranes were incubated overnight at 4° C with primary antibodies diluted 1:1000 in blocking buffer. Membranes were washed 3 times in PBST and incubated with HRP-conjugated secondary antibodies (Cell Signaling Technologies) diluted 1:2000 in PBST for 2 hours at room temperature. Membranes were developed in a 100 mM Tris base solution (pH 8.2) containing hydrogen peroxide, para-coumaric acid, and luminol (all from Biorad). Images were captured on a VersaDocTM MP4000 Molecular Imager (Biorad Laboratories). Primary antibodies used included CD9, BCRT, MDR1, CD81, mTOR, ERK, pAKT, pMAPK, p13AKT, and GAPDH (all from Cell Signaling Technologies).

### ELISA

Blood serum was collected from mice after injection RFP-HCC1806 cells, GFP-BMMSCs, DP-HCC1806:MSCs, and DP-HCC1806:MSCs-siCD9. Similarly, the supernatant was collected from co-cultures, with or without CD9 knockdown in BMMSCs cells, and clarified by centrifugation, first at 500xg for 5 minutes then at 10,000 × g for 10 minutes. The medium was then aliquoted and processed using an ELISA. Levels of inflammatory factors (CXCL12, CCL5) were determined from the serum using commercially available ELISA kits (R&D systems). Prior to use, a frozen aliquot of conditioned medium was thawed on ice and appropriately diluted with buffers recommended by the manufacturer. Optical density (OD) was measured on a plate reader (FLUOstar Omega; BMG Labtech) at an absorbance of 450 nm. Protein concentration was determined after correcting for optical imperfections in the plate by subtracting OD values at 540/570 nm from those obtained at 450 nm.

### HCC1806 growth and drug treatment and preparation

The cell lines HCC1806 were cultured in cultured medium as described above, containing 10% fetal bovine serum (FBS), 1.5 g/L sodium bicarbonate, and 1 mM sodium pyruvate. These cells were maintained in a humidified atmosphere with 5% CO2 at 37° C. Mithramycin A was purchased from Sigma-Aldrich (St. Louis, USA) and reconstituted in DMSO to a final concentration of 100 mM while Doxycycline was purchased from Sigma Aldrich at a stock concentration of 100 mg/mL in DMSO and stored at −20° C. 5-Fluorouracil (Sigma-Aldrich, USA) stock solution was prepared at concentration 50 mg/mL in DMSO, and then diluted 5 times in PBS and stored at −4° C.

### HCC1806 growth assays

Cell growth was determined on RFP-HCC1806 cells, GFP-BMMSCs, and DP-HCC1806:BMMSCs. Cells were seeded into 6-well plates (Corning) at 25,000 cells per well and cultured up to 7 days. On day 7, images were acquired and the cells harvested with trypsin/EDTA for cell counts. For MTT (3-(4, 5-dimethylthiazolyl-2)-2,5-diphenyltetrazolium bromide) (ThermoFisher Scientific), RFP-HCC1806 cells, GFP-BMMSCs, and DP-HCC1806:BMMSCs were seeded into 96-well plates (Corning) at 2,000 cells per well. After 7 days, 20 μL of the MTT solution was added to each well. Plates were incubated at 37° C for 2 h in a humidified atmosphere and 5% CO2. Absorbance was recorded at 490 nm using a plate reader. The number of viable cells was determined using a Cell Counting Kit (BioRad). Cells (3.0 × 10^4^), transfected with siRNA against human CD9 or control RNAs, were seeded on 96-well plates and incubated overnight. After further incubation for 3 days, the kit reagent was added to the medium, and the cells were incubated for 1 h. The absorbance of samples (450 nm) was determined using a scanning multi-well spectrophotometer. For the apoptosis assay, 3.0 × 10^4^cells were seeded on 96-well plates and incubated overnight. After further incubation for 3 days, quantitative viability/cell death in cultured cells were measured using an MTT assay (Roche).

### Breast cancer xenograft model

Female NOD/SCID mice were supplied by Jackson Laboratory (Bar Harbor, ME) and used under a protocol approved by the Institutional Animal Care and Use Committee. RFP-HCC1806 cells (1 × 10^6^ in 100 μL HBSS) were injected into the left mammary fat pad of mice at 12 weeks of age. The RFP-HCC1806 cells were obtained from standard monolayer cultures (experiment 1), from pre-incubated in mice for 4 days cultures (experiment 2), and from BMMSC-CD9 knockout co-cultured BMMSCs:HCC1806 (experiment 6). In addition, animals were injected with 1 × 10^6^ BMMSCs or HCC1806 as a control group. Mice were observed weekly for 8 weeks and were sacrificed after 56 days of tumor cell inoculation. Tumorigenesis was determined via palpation during animal observation and was confirmed by visual assessment of the tumors upon excision. On day 56, animals were euthanized by intraperitoneal injection of ketamine/xylazine. Tumors were excised, photographed, and weighed.

For CD9 knockout experiment 12 6-week-old female NOD/SCID mice were purchased from Jackson Laboraties USA). 1 × 10^6^ DP-HCC1806:BMMSCs-siCD9 cells were implanted into mammary fat pads of the mice. Tumor sizes were measured using Vernier calipers once tumors became palpable. Tumor volumes were calculated using the following equation: tumor volume (cm^3^) = л(length × width^2^)/6. Tumor size was monitored weekly. All mice were sacrificed and tumors were collected for analysis.
